# A cognitive electrophysiological signature differentiates amnestic mild cognitive impairment from normal aging

**DOI:** 10.1186/s13195-016-0229-3

**Published:** 2017-01-19

**Authors:** Juan Li, Lucas S. Broster, Gregory A. Jicha, Nancy B. Munro, Frederick A. Schmitt, Erin Abner, Richard Kryscio, Charles D. Smith, Yang Jiang

**Affiliations:** 1 CAS Key Laboratory of Mental Health, Institute of Psychology, Chinese Academy of Sciences, 100101 Beijing, China; 2 Department of Behavioral Science, University of Kentucky College of Medicine, Lexington, KY 40536 USA; 3 Sanders-Brown Center on Aging, University of Kentucky, Lexington, KY 40536 USA; 4 Department of Neurology, University of Kentucky College of Medicine, Lexington, KY 40536 USA; 5 Oak Ridge National Laboratory, Oak Ridge, TN USA; 6 Department of Epidemiology, University of Kentucky College of Public Health, Lexington, KY 40536 USA; 7 Department of Statistics, University of Kentucky, Lexington, KY 40536 USA; 8 Department of Biostatistics, University of Kentucky College of Public Health, Lexington, KY 40536 USA

**Keywords:** Amnestic mild cognitive impairment, Alzheimer’s disease, Event-related potentials, Working memory, Early detection

## Abstract

**Background:**

Noninvasive and effective biomarkers for early detection of amnestic mild cognitive impairment (aMCI) before measurable changes in behavioral performance remain scarce. Cognitive event-related potentials (ERPs) measure synchronized synaptic neural activity associated with a cognitive event. Loss of synapses is a hallmark of the neuropathology of early Alzheimer’s disease (AD). In the present study, we tested the hypothesis that ERP responses during working memory retrieval discriminate aMCI from cognitively normal controls (NC) matched in age and education.

**Methods:**

Eighteen NC, 17 subjects with aMCI, and 13 subjects with AD performed a delayed match-to-sample task specially designed not only to be easy enough for impaired participants to complete but also to generate comparable performance between subjects with NC and those with aMCI. Scalp electroencephalography, memory accuracy, and reaction times were measured.

**Results:**

Whereas memory performance separated the AD group from the others, the performance of NC and subjects with aMCI was similar. In contrast, left frontal cognitive ERP patterns differentiated subjects with aMCI from NC. Enhanced P3 responses at left frontal sites were associated with nonmatching relative to matching stimuli during working memory tasks in patients with aMCI and AD, but not in NC. The accuracy of discriminating aMCI from NC was 85% by using left frontal match/nonmatch effect combined with nonmatch reaction time.

**Conclusions:**

The left frontal cognitive ERP indicator holds promise as a sensitive, simple, affordable, and noninvasive biomarker for detection of early cognitive impairment.

## Background

Detection of brain changes that precede clinical Alzheimer’s disease (AD) has been a major public health goal owing to the rapidly increasing proportion of the population that is at risk of developing the debilitating illness. Mild cognitive impairment (MCI) is a transitional state between normal aging and dementia. In turn, amnestic mild cognitive impairment (aMCI), a subtype of MCI, has been conceptualized as significant (episodic) memory decline along with relatively preserved global cognition and intact activities of daily living. Individuals with aMCI have a high risk of progression to AD [1]. Neuroimaging biomarkers for early detection of cognitive decline have proven vitally important for emerging interventions that successfully slow progression to AD and for targeting likely MCI/AD converters in clinical trials [2]. However, many of the neuroimaging methods for early detection are expensive, invasive, and require specially trained medical staff [3].

Electroencephalography (EEG), a technique that measures summations of neural postsynaptic potentials at the scalp, has been available for several decades. Averaged EEG signals associated with cognitive events, known as event-related potentials (ERPs), are a well-studied approach for indexing brain mechanisms underlying memory and cognition. Altered ERP signals, either in amplitude or in latency, in patients with AD have also been reported by many groups around the world [4]. Despite this, the science of using cognitive ERPs as biomarkers remains in its infancy.

Recent work has revealed that neurosynaptic changes appear before tau-mediated neuronal injury or brain structure changes and are one of the earliest markers of preclinical AD [5]. Measures of EEG, which directly measures postsynaptic potentials, are sensitive to these early changes and may advance the early detection and diagnosis of “presymptomatic” AD (that is, detection of AD before the appearance of any clinical symptoms whatsoever) [6]. In addition to measuring these early synaptic function changes sensitively, ERP is also less expensive and less invasive than other well-studied early diagnostic biomarkers, such as cerebrospinal fluid (CSF) measurement or imaging with positron emission tomography (PET). Therefore, the primary goal of the present study was to assess the viability of cognitive ERPs as indicators of aMCI.

In addition to the well-known episodic memory impairments, studies have emphasized that working memory and executive function are also affected early in the course of AD [7]. In order to probe working memory deficits, participants were required to perform a version of a delayed match-to-sample (DMS) task that has been used extensively in working memory studies in both primates [8] and humans [9]. In a typical implementation of the DMS task, each trial begins with the presentation of a sample item; then, after a brief delay, items that either match or do not match the sample appear one by one. The participant’s task is to indicate whether each test item matches the prior sample item. Enhanced neural responses in the frontal cortex have been linked to the judgment of whether an item matches the sample in a functional magnetic resonance imaging (fMRI) study [9]. In turn, this judgment has similarly been linked to greater positive P3 component with a scalp distribution maximal at the frontal and central areas that was source-localized to prefrontal and frontal sites in an ERP study [10]; in related studies, this ERP effect showed sensitivity to the course of cognitive aging at frontal sites [11, 12]. Together, these findings implicate a frontal P3 component as an EEG indicator of the working memory status of visually presented items.

Although previous studies have shown that ERP measures can differentiate persons with aMCI from those without impairment [13–21], little is known about whether these measures might capture neural activity differences in the absence of explicit performance impairment. In order to generate and test such a circumstance, we employed an easier version of the DMS task that included fewer test images per trial, fewer blocks, and increased test item presentation time. These steps also allowed participants who had been diagnosed with mild AD to participate in the task, potentially enabling linkage of the neural signatures associated with aMCI to those of AD.

The objectives of the present study were (1) to test whether the frontal ERP signature during working memory differentiates patients with aMCI from normal controls (NC) who are matched in age, education, and performance; (2) to test the progression of the cognitive ERP signature in a group of patients with early AD; and (3) to assess the discrimination accuracy of the ERP signature as a biomarker of disease progression.

## Methods

### Participants

Participants were 18 NC (7 male, 11 female) between the ages of 67 and 83 years (mean = 75.11, SD = 4.95), 16 with aMCI (11 male, 5 female) between the ages of 62 and 90 years (mean = 75.31, SD = 9.21), and 13 patients with AD (5 male, 8 female) between the ages of 66 and 82 years (mean = 75.77, SD = 5.67). The three groups did not differ in age [*F*(2,46) = 0.035, *p* = 0.97], sex distribution [χ^2^(2) = 3.73, *p* = 0.16], or years of education (NC group mean =16.22, SD = 3.02; aMCI group mean = 16.86, SD = 1.96; AD group mean = 17.23, SD = 3.72) [*F*(2,46) = 0.478, *p* = 0.62]. The mean Mini Mental State Examination scores were 29.31 (SD = 0.75, range 28–30) for the NC group, 27.83 (SD = 1.80, range 25–30) for the aMCI group, and 24.44 (SD = 2.76, range 20–29) for the AD group. Mean scores in the AD group were significantly lower than in the NC and aMCI groups (*p* < 0.001).

All participants were community-dwelling individuals who were right-handed and had normal or corrected-to-normal visual acuity. Participants were recruited from the University of Kentucky Alzheimer’s Disease Center (UK-ADC) longitudinal normal volunteer cohort [22, 23]. Inclusion criteria for this cohort are a minimum age of 65 years, cognitive and neurological normality at enrollment, agreement to brain donation to the UK-ADC at death, a designated informant for structured interviews, and willingness to undergo annual examinations. Participants were excluded from the cohort if they had a history of substance abuse, major psychiatric illness, or neurological disease. The annual evaluation includes a comprehensive neuropsychological battery and general physical and neurological examinations that are detailed elsewhere [24, 25].

If any of the following occurs, a cohort participant is evaluated with a more detailed cognitive assessment and formal clinical assessment by study physicians: (1) diagnosis by the examining physician of conversion to MCI or dementia; (2) suspicion of cognitive decline on the part of the supervising neuropsychologist and/or objective decline in the form of annual memory test score 1.5 SD below the previous annual assessment, which is done annually in the UK-ADC consensus conference review [24]; (3) prescription of a cholinesterase inhibitor, *N*-methyl-d-aspartate antagonist, or other treatment associated with the medical diagnosis of dementia by an outside physician; or (4) evidence of functional impairment secondary to cognitive decline from the participant or an informant. The UK-ADC consensus conference reviews these data and a diagnosis of normal, aMCI [1, 26–28], or AD [29] is assigned in accordance with the National Alzheimer’s Coordinating Center’s Uniform Data Set procedures [30]. After conversion to aMCI or AD, each cohort member participated in the present study’s EEG protocols as soon as their scheduling permitted (i.e., most often within 1 month of diagnosis).

EEG and behavioral data analyses performed with a subset of this cohort have been published previously [25, 31, 32]. All participants provided written informed consent before participation. This study was approved by the institutional review board (IRB) of the University of Kentucky.

### Working memory task: DMS task

**Fig. 1:**
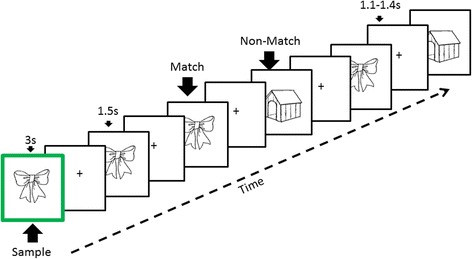
Schematic representing a trial in the delayed match-to-sample task. A sample image with a green border was initially presented for 3 seconds. After a jittered delay (1.1–1.4 seconds), the participant indicated whether each of five successive test images matched or did not match the sample. A new sample image was used in each trial. Individual images (either matching or nonmatching) were tested two or three times per trial. Each working memory (WM) trial lasted approximately 16 seconds. Altogether, 60 trials were performed in 2 blocks of 30 trials each, with a short break between blocks. The working memory task lasted approximately 18 minutes overall

Participants were instructed to memorize a sample image and then indicate whether each of five serially presented objects matched the sample image (Fig. 1) [31]. Stimuli consisted of 120 two-dimensional common objects taken from Snodgrass and Vanderwart [33]. Each picture was presented with a black background and within an area of 8.3 cm × 5.8 cm. All stimuli were presented on a high-resolution color monitor using E-Prime software (Psychology Software Tools, Sharpsburg, PA, USA). Stimuli were presented at a 65-cm visual distance and a visual angle of approximately 7 degrees. Test images were normalized across retrieval status (i.e., matching or nonmatching) for image familiarity and image complexity [33].

### ERP recording

ERP recordings were obtained from 62 scalp sites using Ag/AgCl electrodes embedded in an elastic cap at locations from the extended international 10–20 system. These electrodes were referenced to a midline reference electrode during recording and re-referenced to the average of the right and left mastoid potentials offline. Two additional channels were used for monitoring horizontal and vertical eye movements. Impedance was maintained below 5 kΩ. EEG data were filtered using a band-pass of 0.05–40 Hz and sampled at a rate of 500 Hz. Each averaging epoch lasted 1100 milliseconds, including 100 milliseconds prior to stimulus onset. A regression algorithm implemented with NeuroScan software (Compumedics, Abbotsford, Australia) was used to reduce the influence of blink artifact on the EEG waves. Epochs associated with inaccurate responses or contaminated by electro-ocular artifacts were excluded from analysis.

### Data analysis

Behavioral results were indexed using accuracy and response times (RTs) of correct responses. The accuracy and RT data were subjected to two-way analysis of variance (ANOVA) with the between-subjects factor of group (NC, aMCI, AD) and the within-subjects factor of experimental condition (match, nonmatch).

On the basis of visual inspection of the grand average waveforms and previous studies [13–15], the P3 match vs. nonmatch effect was quantified by calculating the mean amplitude in the 300- to 600-millisecond time window. An omnibus ANOVA was performed over 6 scalp regions involving 18 electrode sites: left frontal (F3, F5, F7), right frontal (F4, F6, F8), left central (C3, F5, T7), right central (C4, C6, T8), left parietal (P3, P5, P7), and right parietal (P4, P6, P8). The factors of this omnibus ANOVA included group (NC, aMCI, AD), experimental condition (match, nonmatch), electrode factors of hemisphere (left, right), anterior-posterior orientation (frontal, central, parietal), and medial-lateral orientation (inferior, middle, superior). Only significant main effects or interactions involving the factors of match/nonmatch and/or group were reported. In addition, planned group comparisons between NC vs. aMCI and aMCI vs. AD on the match vs. nonmatch effects were carried out at the frontal areas. Finally, group discrimination was performed to assess how well the ERP modulation could differentiate aMCI from NC.

Greenhouse-Geisser correction for nonsphericity of data was applied as necessary. The uncorrected degrees of freedom, the corrected *p* values, and the effect sizes (*η*_*p*_^2^) are reported. *p* Values of follow-up pairwise contrasts were adjusted using the Bonferroni correction. For all analyses, the significance level was set to 0.05.

## Results

### Behavioral results

#### Accuracy

Mean accuracy for each group as a function of match/nonmatch are presented in Table 1. ANOVA revealed a main effect of group [*F*(2,44) = 10.99, *p* < 0.001, *η*_*p*_^2^ = 0.33]. Post hoc comparisons revealed that participants with AD performed worse than those in the NC and aMCI groups (*p* < 0.01), but no significant differences were found between the NC and aMCI groups.

**Table 1:** Mean accuracy and mean reaction time (ms) for each response category in three groups (standard deviations of the mean)

Group	Accuracy		RT in milliseconds, mean (SD)	
	Match	Nonmatch	Match	Nonmatch
NC	0.98 (0.16)	0.99 (0.15)	599 (78)	648 (90)
aMCI	0.95 (0.08)	0.98 (0.03)	604 (155)	696 (130)
AD	0.89 (0.10)	0.85 (0.18)	720 (130)	804 (151)

*AD* Alzheimer’s disease, *aMCI* Amnestic mild cognitive impairment, *NC* Normal controls, *RT* response time

#### Response times

Concerning response times (Table 1), ANOVA revealed significant main effects of match/nonmatch [*F*(1,44) = 99.93, *p* < 0.001, *η*_*p*_^2^ = 0.69] and group [*F*(1,2) = 5.35, *p* < 0.01, *η*_*p*_^2^ = 0.20], as well as a two-way match/nonmatch × group interaction [*F*(2,44) = 3.28, *p* < 0.05, *η*_*p*_^2^ = 0.13]. Nonmatch responses were associated with slower RT than responses in the match condition. Participants with AD responded more slowly than those in the NC and MCI groups. The significant match/nonmatch × group interaction reflected these group differences being larger in the nonmatch condition than in the match condition.

#### Summary

Although patients with AD performed more slowly and less accurately than participants in the aMCI and NC groups, the NC and aMCI groups did not differ significantly in either RT or accuracy. These results suggested that the performance between NC and aMCI on the easier task was comparable as planned, whereas patients with dementia performed significantly worse than these groups.

### ERP results

**Fig. 2:**
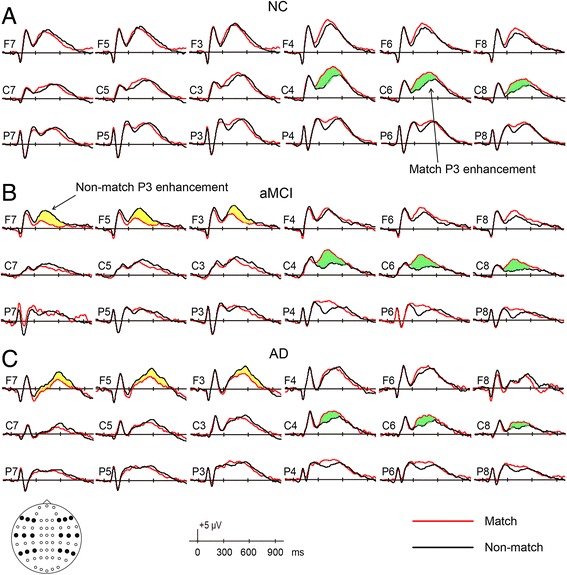
Grand average waveforms elicited by correctly classified match objects and by correctly classified nonmatch objects from −100 milliseconds to +1000 milliseconds in (**a**) the normal control (NC) group, (**b**) the amnestic mild cognitive impairment (aMCI) group, and (**c**) the Alzheimer’s disease (AD) group. Electrode sites are indicated by the inserted montage (F, C, and P stand for frontal, central, and parietal regions on the scalp, respectively). Positive voltages are plotted upward

**Fig. 3:**
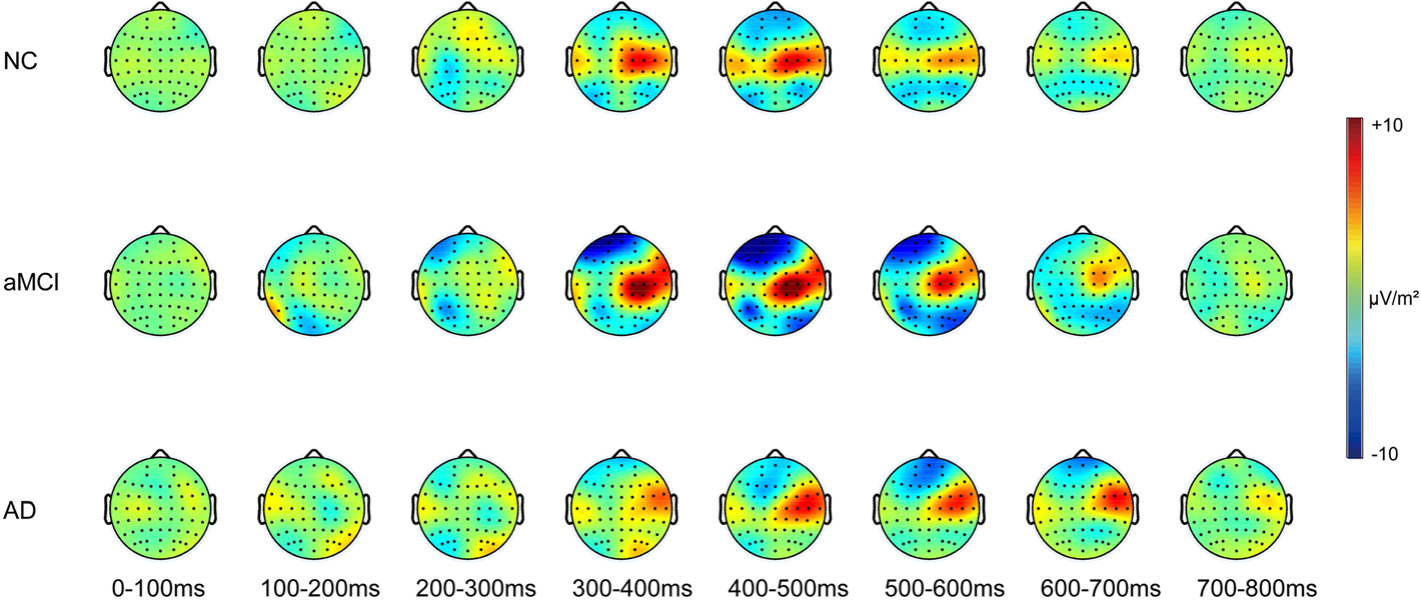
The spatial distribution of current source density (in microvolts per square meter) of match/nonmatch effects (formed by subtracting event-related potentials [ERPs] of nonmatch from ERPs of match) in three groups for each 100 milliseconds within 0- to 800-millisecond latency regions. *AD* Alzheimer’s disease, *aMCI* Amnestic mild cognitive impairment, *NC* Normal controls

The grand average ERPs evoked by correct responses to match and nonmatch objects are shown for all three groups in Fig. 2. The NC group showed the typical P3 match enhancement seen in previous studies [10–12], maximal at right central areas. In addition to the typical P3 match enhancement, a unique and striking feature shown in the aMCI and AD groups was at the left frontal sites, where the nonmatch condition elicited a larger P3. This impression was further verified by evaluating the topographic current source density maps of the difference waveforms by match minus nonmatch (Fig. 3).

#### Omnibus ANOVA

Omnibus ANOVA revealed significant two-way interactions of match/nonmatch × hemisphere [*F*(1,88) = 31.70, *p* < 0.001, *η*_*p*_^2^ = 0.419] and match/nonmatch × anterior-posterior orientation [*F*(2,88) = 13.19, *p* < 0.001, *η*_*p*_^2^ = 0.231], as well as significant three-way interactions of match/nonmatch × hemisphere × group [*F*(2,88) = 3.78, *p* = 0.031, *η*_*p*_^2^ = 0.147] and match/nonmatch × hemisphere × anterior-posterior orientation [*F*(2,88) = 3.83, *p* = 0.040, *η*_*p*_^2^ = 0.080].

Follow-up analyses for the match/nonmatch × hemisphere × anterior-posterior orientation three-way interaction revealed that in the left hemisphere, the amplitudes elicited by the nonmatch condition were larger than the match condition (frontal sites match − nonmatch = 2.791 − 3.784 = −0.993 μV; central sites match − nonmatch = 2.957 − 3.033 = −0.076 μV; parietal sites match − nonmatch = 3.298 − 3.445 = −0.147 μV). The match/nonmatch effect reached significance in the frontal region (*p* = 0.01); in the right hemisphere, more positive-going ERPs for the match condition than for nonmatch were observed, and the match/nonmatch effects reached significance in all three regions, with a trend of being larger at the posterior region than in the anterior region (frontal sites match − nonmatch = 4.263 − 3.656 = 0.607 μV; central sites match − nonmatch = 4.305 − 2.560 = 1.745 μV; parietal sites match − nonmatch = 4.070 − 3.234 = 0.836 μV).

The three-way match/nonmatch × hemisphere × group interaction was further analyzed by exploring the match/nonmatch effect in each hemisphere within each group. In the NC group, the match condition was more positive-going than the nonmatch in both hemispheres, but the differences reached significance only in the right hemisphere (match − nonmatch = 5.468 − 4.447 = 1.021 μV; *p* < 0.001). In the aMCI group, the nonmatch stimuli elicited larger positive amplitudes than the match condition at the left side (match − nonmatch = 1.931 − 2.957 = −1.026 μV, *p* = 0.01), whereas more positive-going ERPs were observed with match than nonmatch at right hemisphere (match − nonmatch = 3.383 − 2.010 = 1.373 μV; *p* < 0.01). The pattern of working memory effects in the AD group presented in a similar way as in the aMCI group, albeit with smaller magnitudes.

#### Planned comparisons

**Fig. 4:**
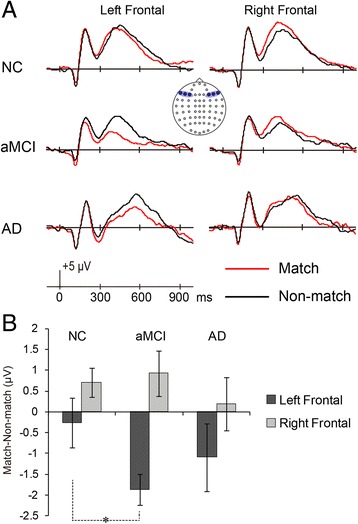
**a** Grand average waveforms from lateral frontal regions elicited by correctly classified match and nonmatch items in three groups. The left frontal (F) region was collapsed across F3, F5, and F7, and the right frontal site was collapsed across F4, F6, and F8. The selected electrode sites are indicated by the *inset*. **b** Mean amplitude of match/nonmatch effects (match − nonmatch) at left and right frontal regions (300–600 milliseconds) according to group. Error bars represent SEM. *Asterisk* denotes significant group difference between NC and aMCI at left frontal region. *AD* Alzheimer’s disease, *aMCI* Amnestic mild cognitive impairment, *NC* Normal controls

Group comparisons on grand average waveforms and mean amplitudes of match/nonmatch effects at frontal region are shown in Fig. 4.

Planned group comparisons were conducted to directly compare groups on the match/nonmatch effect within the frontal sites (i.e., left frontal at F3, F5, and F7; right frontal at F4, F6, and F8). Group × match/nonmatch × hemisphere ANOVA revealed a significant main effect of group [*F*(2,44) = 3.51, *p* < 0.05, *η*_*p*_^2^ = 0.14], a significant two-way hemisphere × match/nonmatch interaction [*F*(1,44) = 26.92, *p* < 0.001, *η*_*p*_^2^ = 0.38], and three-way group × hemisphere × match/nonmatch interaction [*F*(2,44) = 4.28, *p* < 0.05, *η*_*p*_^2^ = 0.16]. In each hemisphere, the planned NC/aMCI and aMCI/AD group comparisons were conducted on the match/nonmatch effect, computed as match − nonmatch. At the left site, the magnitudes of the working memory effect were −0.026 μV in the NC group, −1.865 μV in the aMCI group, and −1.087 μV in the AD group; the difference between NC and aMCI reached significance (*p* < 0.05), whereas the difference between aMCI and AD was not significant. At the right side, the magnitudes of match/nonmatch effect were 0.709 μV in the NC group, 0.926 μV in the aMCI group, and 0.186 μV in the AD group. At the right site, neither of the group comparisons was significant.

#### Summary

In the right hemisphere, a similar P3 match enhancement was found in all three groups, whereas in left frontal areas, a reversed pattern (i.e., P3 nonmatch enhancement) was observed in both the aMCI and AD groups, with a larger such effect seen in the aMCI group.

### Group discrimination

**Fig. 5:**
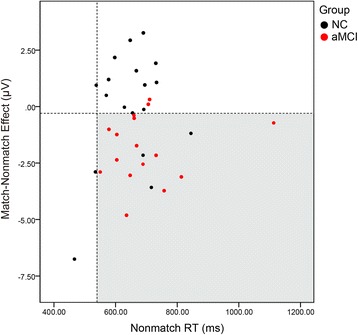
Group discrimination of amnestic mild cognitive impairment (aMCI) from normal controls (NC). Scatterplot of individual subject data for the match/nonmatch effect (mean amplitude difference between 300 and 600 milliseconds at left frontal [F] region collapsed across F7, F5, and F3 for match − nonmatch) and nonmatch reaction times (RTs; mean for correctly identified nonmatch items). The *dashed lines* indicate cutoff values. With match/nonmatch effect less than or equal to −0.39 μV and nonmatch RT greater than or equal to 550 milliseconds, the group discrimination was 85.3%, sensitivity was 87.5%, and specificity was 83.3%

On the basis of a planned comparison finding of significant differences between aMCI and NC at the left frontal region, the match/nonmatch difference (i.e., match − nonmatch) from 300 to 600 milliseconds at the left frontal area collapsed over F7, F5, and F3 was adopted as one factor for discrimination analysis. In addition, given that the response times of NC and aMCI became divergent for the nonmatch condition, nonmatch RT was selected as another factor for discrimination analysis. As shown in Fig. 5, by using −0.39-μV and 550-millisecond cutoffs for match/nonmatch effect and nonmatch RT, respectively, the sensitivity was 14/16 = 87.5%, the specificity was 15/18 = 83.3%, and the group discrimination was (14 + 15)/(16 + 18) = 85.3%.

## Discussion

We tested whether cognitive ERP during a working memory task could discriminate aMCI from normal older adults who were well-matched in age, education, and behavioral performance. We found a left frontal ERP signature associated with working memory to be a potentially effective ERP biomarker indicating cognitive decline before measureable changes in behavioral testing.

### Brain regions involved in DMS task

The network of brain regions is involved during the working memory task, regardless of whether visual objects are trial-unique stimuli. The stimuli for the DMS task trial were unique in the present study. Stimuli consisted of 120 two-dimensional common objects. For each of the 60 trials, a new visual object functioned as the target match. Different objects were used as nontarget/nonmatch distractors. The matches and nonmatches were trial-unique. An object was repeated only within a trial. In a previous fMRI study [9], the small set of 30-plus stimuli were studied by the healthy young participants before fMRI scanning. The stimuli were not unique for memory type or unique for each trial. That is, the same visual item can be a memory target match in one trial and a distractor in the next trial. Thus, working memory status but not familiarity is responsible for the cortical responses. Both prefrontal and temporal cortices (hippocampal and parahippocampal) were involved in the DMS task, with match enhancement dominating in prefrontal cortex (Brodmann areas BA46, BA47, and BA9). These results were consistent with those for monkey physiology. For studies involving older adults and patients, we developed the shorter DMS task for clinical application under either EEG or fMRI environments. As the paradigm used in the present study, healthy older adults participated in a DMS task that used unique stimuli for each trial [34]. A network of cortical regions including medial temporal, parietal and prefrontal cortices were engaged in these DMS tasks.

### Frontal compensation and left frontal ERP signature in aMCI

In the right hemisphere, we found enhanced neural responses for target compared with nonmatch responses in all three groups. In addition, in a previous study, we used a similar DMS paradigm applied to young participants [9]. Likewise, a greater P3 component linked to match condition was reported in the young group. The target-related P3 enhancement could be due to the target stimuli being task-relevant and consequently requiring more attentional resources than nontarget stimuli. It should be noted that in our previous study, the match condition took longer for young participants to respond, whereas in the present study, we found that the reaction time of nonmatch was longer than that of match for three aged groups. Furthermore, although the impaired groups’ responses were generally slower, compared with match condition, under the nonmatch condition the group differences tended to become larger. By combining the results from both our previous study and the present study, we concluded that rejecting a distractor became difficult when people got older and even more difficult when MCI appeared during old age, so that the MCI evoked larger left frontal responses of nonmatch vs. match as a compensation mechanism to counteract their impaired ability to inhibit the distractors. As the disease further progressed, a disruption of compensation was detected in AD, which was linked to their reduced performance. These findings were consistent with literature showing that persons with MCI recruit more neural resources than persons without impairment in various cognitive tasks, including memory and attention, to try to compensate for their cognitive deficits [35].

We found that this left frontal ERP signature, together with the nonmatch RT in a DMS task, provided a discrimination accuracy of 85%. Given that this task took only about 18 minutes and EEG recording is a method less expensive and/or less invasive than some other biomarkers, such as magnetic resonance imaging, PET, and CSF sampling, we suggest that this cognitive ERP marker may serve as a dynamic biomarker that could be used before explicit changes in memory performance are measurable or structural changes are seen in the brain.

### Early AD pathology and ERPs of synaptic dysfunction

One of the hallmarks of early AD pathology is synaptic loss in the hippocampus and neocortex at the medial temporal cortices in persons with MCI or AD [36]. Previous published neuropathological work on other participants in the UK-ADC longitudinal cohort has implicated loss of afferents from the entorhinal cortex to frontal and parietal regions in the progress of AD [37, 38]. Such afferents are known to be implicated in the instantiation of working memory through short-term synaptic facilitation, and the working memory task used in the present study is known to incorporate prefrontal resources as task difficulty increases through incorporation of additional distractors [39, 40]. Thus, we suggest that the left frontal match/nonmatch effect has good face validity as a cognitive biomarker of early AD and reflects cognitive compensation for the nonmatch working memory condition, which is associated with greater difficulty in the early course of clinical AD [31].

### Future directions

This study was limited by its small sample size and cross-sectional nature. In future work, we aim to validate the current ERP biomarker with a larger sample size. In addition, we will follow the NC and aMCI individuals to confirm the validity of this simple and noninvasive biomarker through within-subject conversions. Because the participants in the present study were UK-ADC volunteers who have agreed to donate their brains for postmortem pathological assessment, the relationship of the cognitive ERPs found in this and future studies to the neuropathological correlates of aMCI and AD will be also validated after autopsy.

## Conclusions

By use of a DMS task, we found a unique P3 nonmatch enhancement effect in persons with aMCI despite comparable memory performance on this task relative to the NC group. We suggest that this indexes compensation for impaired rejecting of distractor stimuli in aMCI and that disruption of this compensation mechanism in AD was linked to their reduced performances. This left frontal ERP modulation provides a sensitive, noninvasive, and less expensive neurosignature for early detection of aMCI.

## Abbreviations

AD: Alzheimer’s disease
aMCI: Amnestic mild cognitive impairment
ANOVA: Analysis of variance
BA: Brodmann area
CSF: Cerebrospinal fluid
DMS: Delayed match-to-sample task
EEG: Electroencephalography
ERP: Event-related potential
fMRI: Functional magnetic resonance imaging
IRB: Institutional review board
MCI: Mild cognitive impairment
NC: Normal controls
PET: Positron emission tomography
RT: Response time
UK-ADC: University of Kentucky Alzheimer’s Disease Center
